# Ectopic BH3-Only Protein Bim Associates with Hsp70 to Regulate Yeast Mitophagy

**DOI:** 10.1134/S1607672923700485

**Published:** 2023-12-13

**Authors:** Linjie Yuan, Yuying Wang, B. A. Margulis, Ting Song, Ziqian Wang, Zhichao Zhang

**Affiliations:** 1 School of Chemistry, Dalian University of Technology, Dalian, China; 2 School of Life Science and Technology, Dalian University of Technology, Dalian, China; 3 Institute of Cytology, Russian Academy of Sciences, St. Petersburg, Russia

**Keywords:** B-cell lymphoma 2 family, Bim, Hsp70, mitophagy, yeast

## Abstract

Mitophagy, a form of selective autophagy, plays an essential role to maintain a population of healthy and functional mitochondria for normal cellular metabolism. Acting mainly as one of the B-cell lymphoma 2 (Bcl-2) family pro-apoptotic members, Bim (also known as BCL2L11) was identified to be a co-chaperone of Hsp70 to promote mitophagy in mammalian cells. Herein, with the help of a specific Hsp70/Bim disruptor and Om45-GFP processing assay, we illustrated that ectopic BimEL is able to promote mitophagy through Hsp70/Bim interaction in yeast, where Bax/Bak is absent. The Hsp70/Bim-mediated mitophagy is conserved in eukaryotes, from yeast to humans.

## INTRODUCTION

Autophagy is a catabolic process that non-selectively degrades cytoplasmic components and organelles, and is conserved in almost all eukaryotes [1–4]. The inhibition of stress-induced autophagy has been proven to be promising for cancer therapy [5] because the level of autophagy can be dramatically augmented when mammalian cells encounter environmental stresses, including tumorigenic environment stress, and anticancer drug treatment. In contrast to autophagy, selective autophagy can degrade specific organelles or proteins [6]. Mitophagy, for example, ensuring the selective removal of damaged or dysfunctional mitochondria and degradation in lysosomes/vacuoles, represents a flexible mechanism supporting the metabolic adaptation and survival of cancer cells within the harsh tumor microenvironment [7–9]. In another aspect, mitochondrial dysfunction has been associated with aging and various human diseases, such as neurodegeneration, metabolic disorders, diabetes, and cancer [10]. Mitophagy is a process that maintains a population of healthy and functional mitochondria for normal cellular metabolism.

In 2020 our group found Bim, which is a BH3-only protein, is a co-chaperone of Hsp70 [11]. Very recently, a new mitophagy pathway in mammalian cells was unveiled to be mediated by Hsp70/Bim dimer induced TOMM20 ubiquitylation [12]. Of note, it is a Bax/Bak independent mitophagy pathway. Both mitophagy and apoptosis occur in mitochondrial and they share many components such as Bcl-2 family members [13]. The crosstalk between the two pathways, apoptosis and mitophagy, remains largely elusive since they always interfere with each other. For example, some studies using small molecule Bcl-2 inhibitors resulted in different conclusions [14–16]. It is still confusing whether or not Bax/Bak is needed in mitophagy.

It has been demonstrated that selective degradation of mitochondria by mitophagy could occur in the yeast *S. cerevisiae*, thus providing a useful model to understand the molecular mechanism, actors and regulation of this process [17, 18]. The process of mitophagy is believed less complex in the yeast *S. cerevisiae* than in mammals since much fewer proteins there, e.g., cononical Bcl-2 family members are absent. Additionally, we have identified the in vitro positive co-chaperone activity of Bim could translate into the yeast growth promotion and help Hsp70 to fold Ras-like protein in yeast through Hsp70/Bim dimer [19].

Herein, we investigated the Hsp70/Bim mediated mitophagy in yeast since there is not any killer of Bcl-2 family, and then no apoptosis could interfere with the cell’s fate upon ectopic BimEL overexpressed. With the help of an Hsp70 inhibitor, **S1g-6** [20] developed by our group that exhibits sub-µM binding affinity towards Hsp70 and selectively disrupts Hsp70-Bim protein-protein interaction (PPI) both in vitro and in situ, we identified ectopic BimEL can promote mitophagy through Hsp70/Bim interaction in yeast, where Bax/Bak is absent.

## MATERIALS AND METHODS

### Yeast Strains, Plasmids, and Growth Conditions

The yeast strain used in this study is INVSc1(MATα his3Δ1 leu2 trp1-289 ura3-52; Shanghai Weidi Biotechnology, Shanghai, China, YC1050). Yeast cells were grown in rich medium (YPD; 1% yeast extract, 2% peptone, 2% glucose), lactate medium (YPL; 1% yeast extract, 2% peptone, 2% lactate, pH 5.5). Starvation experiments were performed in a synthetic minimal medium lacking nitrogen (SD-N; 0.17% yeast nitrogen base without amino acids, 2% glucose). Synthetic dextrose minimal medium lacking uracil medium (SD-Ura; 0.67% yeast nitrogen base, 2% glucose, 0.01% leucine, 0.002% Histidine, 0.002% Tryptophan) was applied to screen strains expressing plasmids. The yeast strain was transformed with the plasmids pYES2, pYES2-BimEL, and pYES2-Hsp70. The plasmid pYES2 as vector control, the plasmid pYES2-BimEL carrying human BimEL cDNA (the sequences encoding amino acids (aa) 1 to 198 of human BimEL) and the plasmid pYES2-Hsp70 cDNA (the sequences encoding amino acids (aa) 1 to 646 of human Hsp70) were purchased from Miaolingbio (Wuhan, Hubei, China). Transformations were performed using the PEG–lithium acetate method.

### Strain Construction

Yeast expressing chimeric Om45-GFP was generated by integrating a DNA fragment encoding GFP at the 3′ end of endogenous Om45 using a PCR-based integration method [21]. A DNA fragment enco-ding  GFP with a selective marker is PCR-amplified using pPICZα A-Om45 PS-linker-GFP(S65T) as    a    template plasmid and primers (5′-GGCCCAAAAGAAGTACGATGAAGCGTTGAAAAAGTACGATGAAGCCAAGAACAAATTC, 5′-GAGAAACATGTGAATATGTATATATGTTATGCGGGAACCACTCACATGTTGGTCTCCAG, and 5′-CAGCAAAGACCATATGTGATTTCAAATATAAAAAATGAGAAACATGTGAATATGTATA).

### Om45-GFP Processing Mitophagy Assay

To monitor mitophagy, the Om45-GFP processing assay was adapted from Kanki et al. [22, 23]. Briefly, induction of mitophagy by nitrogen starvation: cells expressing Om45-GFP were cultured in YPD to mid-log phase, then the cells were cultured in YPL for 14 h to amplify mitochondria, and finally shifted to SD-N for 6 h to induce mitophagy.

### Yeast Growth for Heat Stress Experiments

Yeast cells were cultivated in liquid YPD medium with rapid agitation at 30°C overnight. Cells were grown in fresh YPL at 30 and 37°C with a starting OD_600_ of 0.2, and grown for 24 h. Cell pellets were then collected and harvested for experimentation.

### Antibodies and Reagents

Antibodies used in this study were: mouse anti-GFP (ABT2020; Abbkine), mouse anti-GAPDH (ABL1020; Abbkine), rabbit anti-Bim (#2933; Cell Signaling Technology) and mouse anti-Hsp70 (#22190518; Sigma-Aldrich). **S1g-6** (*6-(cyclohexylthio)-3-((2-morpholinoethyl)amino)-1-oxo-1H-phenalene-2-carbonitrile*) and **S1** (*1-Oxo-6-thiomorpholino-1H-phenalene-2,3-dicarbonitrile*) were synthesized as previously described [20, 24, 25] and dissolved to a final concentration of 10 mM in dimethyl sulfoxide (DMSO) and stored at –20°C. Chloroquine (CQ; S6999) was purchased from Selleckchem and dissolved in DMSO at a final concentration of 200 mM, stored at –20°C.

### Preparation of Protein Extracts and Western Blotting

Immediately after harvesting the cell aliquots, 10% trichloroacetic acid was added. Then, the samples were placed on ice for 1 h. Pellet the proteins by centrifugation, and discard the supernatant by aspiration. The protein was pelleted twice by adding 1 mL of ice-cold acetone and re-pelleted by centrifugation. Air-dry the pellets. The air-dried cell pellet was resuspended in sample buffer and vortexed with an equal volume of acid-washed glass beads for 2 min each time, followed by 5 times vortex. The samples were incubated at 100°C for 5 min.

The Protein samples were separated on 12% SDS-PAGE and transferred to PVDF membranes at 350 mA, for 60 min in 1× Transfer buffer (200 mM Glycine, 20 mM Tris, 20% methanol). The membranes were blocked with Blocking buffer (5 vol % non-fat dried milk) at room temperature for 1 h. After blocking, membranes were incubated overnight with the corresponding antibody at 4°C and washed three times with PBST (PBS with 1 vol % Tween-20). Then membranes were incubated with secondary antibodies coupled to horseradish peroxidase at room temperature for 2 h. Finally, the blots were developed with SuperSignal West Dura Kit (Thermo Scientific).

### Co-immunoprecipitation (Co-IP)

Yeast cells were lysed with Yeast Total Protein Extraction Kit (Sangon Biotech, Shanghai, China, C500013), according to the manufacturer’s instructions. Whole-cell lysates were obtained and normalized by BSA assay. Antibody (2 μg) was added to an input volume of 500 μL containing (1–3) mg/mL protein. After incubating overnight at 4°C, 40 μL of protein A + G Agarose (Beyotime Biotechnology, Shanghai, China, P2012) were added and then incubated for 2 h at 4°C with constant rotation. Protein A-Sepharose beads containing the immunoprecipitate were washed three times with lysed buffer supplemented with 1% protease inhibitor, resuspended in 1× loading buffer (Beyotime Biotechnology, Shanghai, China, P0015L), and boiled for 15 min by immunoblotting analysis.

### Fluorescence Microscopy

OLYMPUS FV-3000 laser scanning confocal microscopy with a 100× objective lens was used in order to conduct the fluorescence imaging. GFP was excited at 488 nm, and the emission spectra were collected at 510−550 nm. Fluorescence imaging of all groups were performed under the same experimental conditions. Image analysis was performed using FV10-ASW 3.0.

### Statistical Analysis

Unless otherwise stated, western blot data were quantified using ImageJ software to measure the relative intensity of each band. All quantification data were presented as the mean ± SD (standard deviation) from at least 3 independent experiments. Student’s *t*-test was used for statistical significance. A value of *p* < 0.05 was considered significant.

## RESULTS

### BimEL Promotes Mitophagy in Yeast Cells

**Fig. 1.**
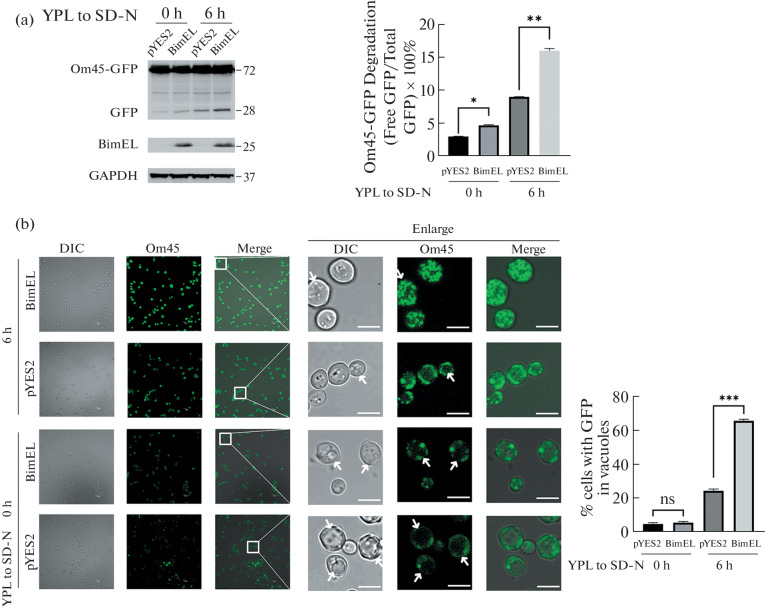
Effect of Bim on mitophagy in yeast. (a) Strains expressing Om45-GFP transfected with pYES2-BimEL and pYES2 plasmids were cultured in YPL medium until mid-log growth, then shifted to SD-N medium for 0 and 6 h. GFP processing was monitored by immunoblotting with anti-GFP and anti-GAPDH (loading control) antibodies. Quantification of the percentage of GFP degraded from Om45-GFP bands: % Om45-GFP Degradation = Density of GFP band/(Density of Om45-GFP band + Density of GFP band) × 100%. (b) Cells were observed by laser scanning confocal microscopy. The percentage of cells with GFP in vacuoles was quantified and presented. Arrows indicate vacuoles. DIC, differential interference contrast. Scale bars, 5 μm. The pictures containing more than 60 cells in each group were used for statistical analysis. NS indicates no significant, * indicates *p* < 0.05, ** indicates *p* < 0.01, *** indicates *p* < 0.001; mean ± SD; *n* = 3.

To investigate the effect of Bim on mitophagy of yeast, we generated yeast cells overexpressing BimEL and examined its effects on mitophagy by using the Om45-GFP processing assay [22]. The strains transformed empty vector pYES2 was used as a control. Om45-GFP is a mitochondrial outer membrane protein. Following autophagy of mitochondria, Om45-GFP is degraded, releasing the intact form of GFP. Therefore, the level of mitophagy could be semi-quantitatively monitored by measuring using immunoblotting the amount of GFP (MW:28) processed from Om45-GFP(MW:72). As shown in Fig. 1a, the level of GFP processed from Om45-GFP was increased when cells were cultured in lactate medium (YPL) and then shifted to nitrogen starvation medium (SD-N) for 6 h compared to that for 0 h, consistent with previous reports. Further, about twice release of GFP from Om45-GFP occurred in strains overexpressed BimEL (*p* < 0.01, Fig. 1a, right). Next, we used fluorescence microscopy to detect the increased intensity and accumulation of GFP fluorescence in the vacuole lumen, which reflected the level of mitophagy. Consistent with the immunoblotting assay, our GFP fluorescence images showed that nitrogen starvation induced an accumulation of vacuolar GFP signals as indicated by increased intensity of bright green fluorescence after 6 h incubation in SD-N medium, and the strongest fluorescence appeared in BimEL expressing yeast. Moreover, overexpression of BimEL significantly enhanced the fluorescence accumulation in the vacuole lumen (*p* < 0.01, Fig. 1b). Additionally, BimEL expression promoted fragmented vacuole formation as shown by fluorescence, which is always found in mitophagy rather than non-selective autophagy [26, 27]. Taken together, these data showed that BimEL acts as a positive regulator of mitophagy in yeast.

### Heat Stress Enhances Bim-induced Mitophagy

**Fig. 2.**
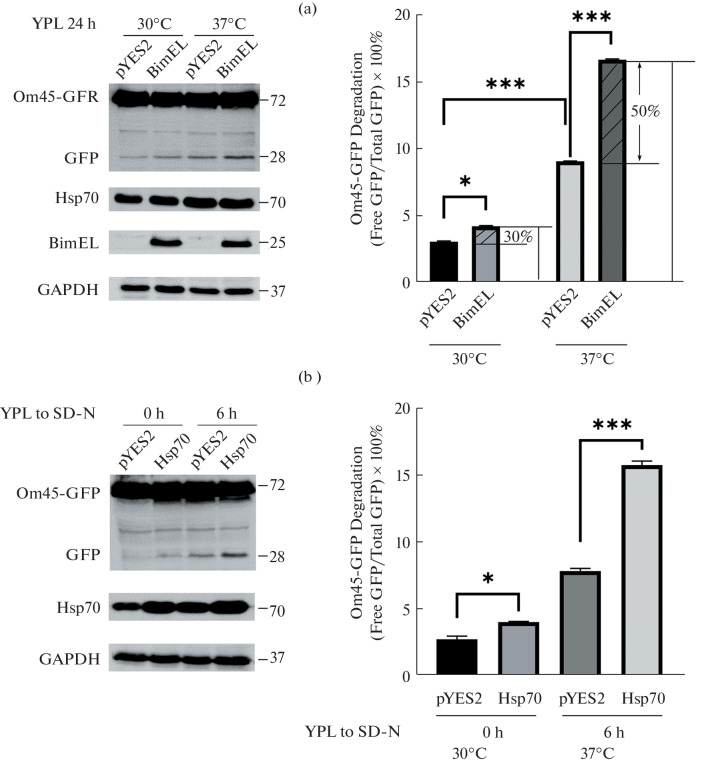
Effect of heat stress on Bim-induced mitophagy in yeast. (a) Strains expressing Om45-GFP transfected with pYES2-BimEL and pYES2 plasmids were cultivated in YPD medium at 30°C overnight. Then cells were shifted to YPL medium and grown at 30 and 37°C for 24 h, respectively. (b) Strains expressing Om45-GFP transfected with pYES2-Hsp70 and pYES2 plasmids were cultured in YPL medium until mid-log growth, then shifted to SD-N medium for 0 and 6 h. GFP processing was monitored by immunoblotting with anti-GFP and anti-GAPDH (loading control) antibodies. GFP processing was monitored by immunoblotting with anti-GFP, and anti-GAPDH (loading control) antibodies. Quantification of the percentage of GFP degraded from Om45-GFP bands: % Om45-GFP Degradation = Density of GFP band/(Density of Om45-GFP band + Density of GFP band) × 100%. * Indicates *p* < 0.05, ** indicates *p* < 0.01, *** indicates *p* < 0.001; mean ± SD; *n* = 3.

We have identified BimEL could interact with Hsp70 in yeast and positively regulate Hsp70 ATPase activity previously [19]. Moreover, Hsp70/Bim dimers are able to rescue yeast from heat shock. Heat stress induces mitochondrial dysfunction and activates mitochondrial stress responses, including mitophagy. Hereto we tested the behavior of BimEL under heat stress and, more importantly, we investigated the contribution of Hsp70 protein to yeast mitophagy because it could be upregulated in heat shock. We treated pYES2-BimEL transformed yeast cells at physiological 30 and 37°C, respectively. Empty vector pYES2 transformed cells were treated and assayed in parallel as the control. Compared to the cells cultured in YPL (respiratory growth) at 30°C for 24 h, the level of GFP processed from Om45-GFP was increased as an indicator of mitophagy induction after being cultured in YPL at 37°C (Fig. 2a).

Moreover, we compared the effect of BimEL on mitophagy between cells culture at 30 and 37°C. Firstly, when Hsp70 was upregulated under heat stress, mitophagy was evoked even without BimEL expression as indicated by processed GFP increased from less than 5 to about 10% (Fig. 2a, right panel). This suggested Hsp70 itself is involved in heat stress-induced mitophagy. Interestingly, we found that at 30°C, overexpression of BimEL displayed a moderate effect on mitophagy, as shown by about 30% increase of processed GFP (*p* < 0.01, Fig. 2a, right panel). Under conditions of heat shock at 37°C, overexpression of BimEL led to about 50% increase in processed GFP. The data showed that BimEL contributed more and more to mitophagy as long as the level of Hsp70 was increasing.

However, heat stress causes various metabolic changes in addition to increased expression of Hsp70. Therefore, we generated yeast cells overexpressing Hsp70 and examined its effects on mitophagy by using the Om45-GFP processing assay, as shown in Fig. 2b. When cells were cultured in lactate medium (YPL) and then shifted to nitrogen starvation medium (SD-N) for 0 h, the effect of Hsp70 overexpression on mitophagy was essentially the same in yeast cells as in the control group. The level of GFP processed from Om45-GFP was increased when cells were cultured in nitrogen starvation medium (SD-N) for 6 h, and yeast cells overexpressing Hsp70 greatly promoted the release of GFP. Our results illustrate that overexpression of Hsp70 alone has a promoting effect on mitophagy.

### BimEL Promotes Mitophagy through Hsp70/Bim Interaction

**Fig. 3.**
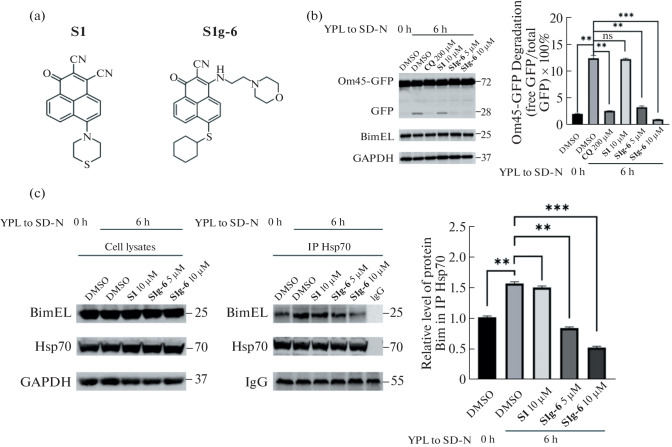
Effects of small molecules on mitophagy in yeast. (a) The structures of **S1** and **S1g-6**. (b) Strains expressing Om45-GFP transfected with pYES2-BimEL were cultured in YPL medium to the mid-log growth phase, then shifted to SD-N medium with 200 μM **CQ**, 10 μM **S1**, 5 and 10 μM **S1g-6**, respectively for 6 h, DMSO was added as a control. GFP processing was monitored by immunoblotting with anti-GFP, and anti-GAPDH (loading control) antibodies. Quantification of the percentage of GFP degraded from Om45-GFP bands: % Om45-GFP Degradation = Density of GFP band/(Density of Om45-GFP band + Density of GFP band) × 100%. (c) Western blot and Co-IP analysis of Hsp70 and Bim levels in the pYES2-BimEL strain according to the above treatment conditions. Hsp70 was co-immunoprecipitated and Bim was determined by western blotting. NS indicates no significant, ** indicates *p* < 0.01, *** indicates *p* < 0.001; mean ± SD; *n* = 3.

When we verified either Hsp70 or BimEL could promote mitophagy, we continued to investigate the role of Hsp70/Bim dimer in yeast mitophagy. Two analogs were chosen as probes. **S1g-6** [20], specifically targets Hsp70-Bim protein–protein interaction (PPI) while **S1** [24, 25] specifically disrupts Bcl-2-Bim PPI (Fig. 3a). We cultured pYES2-BimEL transformed yeast cells in YPL and then shifted to SD-N in the presence of the two analogs, respectively. An equivalent of DMSO was added as vehicle control. Chloroquine (**CQ**, an inhibitor of autophagy-lysosome pathway) was used as a positive control. The mitophagy was measured by GFP processing.

As shown in Fig. 3b, 200 μM **CQ** significantly decreased the level of GFP processed from Om45-GFP [28], consistent with previous reports. **S1g-6** at the dose of 5 and 10 μM led to a dose-dependent decrease in the amount of processed GFP, indicating that **S1g-6** inhibited mitophagy. In contrast, 10 μM **S1** exhibited no effect on autophagic flux. Given the mitophagy-inhibitory effect of **S1g-6**, we suspected that BimEL promotes mitophagy through Hsp70/Bim interactions in yeast cells, as similar as it did in mammalian cells.

To test it, we performed co-immunoprecipitation (Co-IP) experiments. Cell lysates were immunoprecipitated with Hsp70 antibody and then immunoblotted for Hsp70 and Bim. As shown in Fig. 3c, we found that the amount of Bim that coimmunoprecipitates with Hsp70 was increased by about 1.5-fold in pYES2-BimEL transformed yeast upon nitrogen starvation. **S1g-6** at the concentration of 5 and 10 μM, respectively, led to a dose-dependent disruption of the Hsp70/Bim interaction, which was paralleled by a dose-dependent decrease in the amount of processed GFP. **S1** does not affect Hsp70/Bim interaction.

Taken together, these results demonstrated that BimEL promotes mitophagy through Hsp70/Bim interaction. Hsp70/Bim PPI is the target responsible for the mitophagy-inhibiting activity of **S1g-6**.

## DISCUSSION

In the past decades, increasing evidence has shown that the functions of Hsp70 through their protein-protein interactions (PPIs) with a subset of co-chaperones are of interest for cancer specific functions. Since BH3-only protein Bim was found by our group to be a co-chaperone of Hsp70, the Hsp70-Bim PPI actions in both apoptosis and autophagy have been intensively investigated. Most recently, Hsp70/Bim dimer was illustrated as a mitophagy player in mammalian cells independently of Bax/Bak. Herein, we tried to find if this is a conserved way of mitophagy regulation in yeast since we have identified that ectopic BimEL is able to reconstruct its co-chaperone function in yeast.

Firstly, a mitophagy specific induction was applied that cells were cultured in YPL to stimulate mitochondria and then shifted to nitrogen starvation medium for autophagy occurrence. By using Om45 tagged with GFP (Om45-GFP) which is anchored in the mitochondrial surface and delivered to the vacuole via mitophagy, BimEL was identified to promote mitophagy via fluorescence microscopy and immunoblotting. Secondly, upregulation of Hsp70 was also found to promote mitophagy in yeast cells cultured in YPL for 24 h. It has been identified that BimEL can protect yeast from heat shock through positive regulation of the ATPase activity of Hsp70. Here, another mechanism underlying its protection was unveiled. Moreover, Hsp70 was demonstrated to play a role in yeast mitophagy for the first time. Since there are no Bcl-2 family effectors, Bax and Bak in yeast, the Bax/Bak independent manner of Hsp70/Bim mediated mitophagy was re-identified. Supported by the involvement of de-ubiquitination system in yeast mitophagy [29], it is likely that ubiquitous system is involved in Hsp70/Bim mediated yeast mitophagy as it is in mammalian cells. A diverse siRNA screening strategy validated HSPA1L (Hsp70 member) regulates parkin translocation in mammalian cells, while Bim was also included in the strong candidates that its siRNA affected parkin translocation to the same degree as HSPA1L [30]. We had then illustrated that Bim positively regulates parkin as a co-chaperone of Hsp70 in mammalian cells before this study. Herein, our results suggested the existence of unknown E3 ubiquitin ligase and substrate in yeast since there is no parkin.

When Hsp70 or Bim was identified to promote mitophagy alone, the progressive promotion of mitophagy of Bim under heat shock supported the cooperation of Bim with Hsp70. Consistently, small molecule Hsp70/Bim inhibitor **S1g-6** identified directly that Hsp70/Bim PPI mediates yeast mitophagy.

The very newly found mitophagy regulation by Hsp70/Bim in mammalian cells was identified in yeast, enhancing the understanding of Hsp70 physiological and pathological functions. It has been argued for decades if Hsp70 could be an anti-cancer target although it is upregulated in many cancers and continues to be upregulated in anticancer treatment [31]. Autophagy itself has become a target for cancer therapy because it is one of the most important cell responses under stressful conditions, including intrinsic oncogenes and extra stimuli from anticancer agents. The understanding of Hsp70 associated Bim to regulate selective autophagy, mitophagy is a step to reveal the significance of Hsp70 as an autophagy-related anti-cancer target.

## References

[1] Hirota Y., Kang D., Kanki T. (2012). The physiological role of mitophagy: New insights into phosphorylation events. Int. J. Cell Biol.

[2] Li W., He P., Huang Y., Li Y.F., Lu J., Li M., Kurihara H., Luo Z., Meng T., Onishi M. (2020). Selective autophagy of intracellular organelles: recent research advances. Theranostics.

[3] Klionsky D.J. (2005). The molecular machinery of autophagy: unanswered questions. J. Cell Sci.

[4] Chen Y.Q., Klionsky D.J. (2011). The regulation of autophagy—unanswered questions. J. Cell Sci.

[5] Das C.K., Mandal M., Kögel D. (2018). Pro-survival autophagy and cancer cell resistance to therapy. Cancer Metastasis Rev.

[6] Fimia G.M., Kroemer G., Piacentini M. (2013). Molecular mechanisms of selective autophagy. Cell Death Differ.

[7] Yun C.W., Lee S.H. (2018). The roles of autophagy in cancer. Int. J. Mol. Sci.

[8] Lorin S., Hamaï A., Mehrpour M., Codogno P. (2013). Autophagy regulation and its role in cancer. Semin. Cancer Biol.

[9] Vara-Perez M., Felipe-Abrio B., Agostinis P. (2019). Mitophagy in cancer: a tale of adaptation. Cells.

[10] Wallace D.C. (2005). A mitochondrial paradigm of metabolic and degenerative diseases, aging, and cancer: a dawn for evolutionary medicine. Annu. Rev. Genet.

[11] Guo Z., Song T., Wang Z., Lin D., Cao K., Liu P., Feng Y., Zhang X., Wang P., Yin F. (2020). The chaperone Hsp70 is a BH3 receptor activated by the pro-apoptotic Bim to stabilize anti-apoptotic clients. J. Biol. Chem.

[12] Song T., Yin F., Wang Z., Zhang H., Liu P., Guo Y., Tang Y., Zhang Z. (2023). Hsp70-Bim interaction facilitates mitophagy by recruiting parkin and TOMM20 into a complex. Cell. Mol. Biol. Lett.

[13] Levine B., Sinha S., Kroemer G. (2008). Bcl-2 family members: dual regulators of apoptosis and autophagy. Autophagy.

[14] Sorice M. (2022). Crosstalk of Autophagy and Apoptosis. Cells.

[15] Nikoletopoulou V., Markaki M., Palikaras K., Tavernarakis N. (2013). Crosstalk between apoptosis, necrosis, and autophagy, *Biochim. Biophys. Acta, Mol.*. Cell Res.

[16] Maiuri M.C., Zalckvar E., Kimchi A., Kroemer G. (2007). Self-eating and self-killing: Crosstalk between autophagy and apoptosis. Nat. Rev. Mol. Cell Biol.

[17] Bhatia-Kissova I., Camougrand N. (2021). Mitophagy in yeast: decades of research. Cells.

[18] Innokentev A., Kanki T. (2021). Mitophagy in yeast: molecular mechanism and regulation. Cells.

[19] Pan H., Song T., Wang Z., Guo Y., Zhang H., Ji T., Cao K., Zhang Z. (2021). Ectopic BH3-only protein Bim acts as a cochaperone to positively regulate Hsp70 in yeast. J. Biochem.

[20] Wang Z., Song T., Guo Z., Uwituze L.B., Guo Y., Zhang H., Wang H., Zhang X., Pan H., Ji T. (2021). A novel Hsp70 inhibitor specifically targeting the cancer-related Hsp70-Bim protein-protein interaction. Eur. J. Med. Chem.

[21] Longtine M.S., McKenzie A., Demarini D.J., Shah N.G., Wach A., Brachat A., Philippsen P., Pringle J.R. (1998). Additional modules for versatile and economical PCR-based gene deletion and modification in *Saccharomyces cerevisiae*. Yeast.

[22] Kanki T., Kang D., Klionsky D.J. (2009). Monitoring mitophagy in yeast: the Om45-GFP processing assay. Autophagy.

[23] Kanki T., Okamoto K. (2014). Assays for autophagy ii: Mitochondrial autophagy. Methods Mol. Biol.

[24] Zhang Z., Song T., Zhang T., Gao J., Wu G., An L., Du G. (2011). A novel BH3 mimetic S1 potently induces Bax/Bak-dependent apoptosis by targeting both Bcl-2 and Mcl-1. Int. J. Cancer.

[25] Zhang Z., Wu G., Xie F., Song T., Chang X. (2011). 3-Thiomorpholin-8-oxo-8H-acenaphtho[1,2-b]pyrrole-9-carbonitrile (S1) based molecules as potent, dual inhibitors of B-cell lymphoma 2 (Bcl-2) and myeloid cell leukemia sequence 1 (Mcl-1): Structure-based design and structure-activity relationship studies. J. Med. Chem.

[26] Kiššova I., Salin B., Schaeffer J., Bhatia S., Manon S., Camougrand N. (2007). Selective and non-selective autophagic degradation of mitochondria in yeast. Autophagy.

[27] Wiemken A., Matile P., Moor H. (1970). Vacuolar dynamics in synchronously budding yeast. Arch. Mikrobiol.

[28] Wen F.P., Guo Y.S., Hu Y., Liu W.X., Wang Q., Wang Y.T., Yu H.Y., Tang C.M., Yang J., Zhou T. (2016). Distinct temporal requirements for autophagy and the proteasome in yeast meiosis. Autophagy.

[29] Müller M., Kötter P., Behrendt C., Walter E., Scheckhuber C.Q., Entian K.D., Reichert A.S. (2015). Synthetic quantitative array technology identifies the Ubp3-Bre5 deubiquitinase complex as a negative regulator of mitophagy. Cell Rep.

[30] Hasson S.A., Kane L.A., Yamano K., Huang C.H., Sliter D.A., Buehler E., Wang C., Heman-Ackah S.M., Hessa T., Guha R. (2013). High-content genome-wide RNAi screens identify regulators of parkin upstream of mitophagy. Nature.

[31] Murphy M.E. (2013). The HSP70 family and cancer. Carcinogenesis.

